# Supplementary material for: Contrast and luminance adaptation alter neuronal coding and perception of stimulus orientation
Source: Nat Commun. 2019 Feb 26;10:941. doi: 10.1038/s41467-019-08894-8 (PMC6391449; doi:10.1038/s41467-019-08894-8)
Supplement: Supplementary file 1 — Supplementary Information [file 41467_2019_8894_MOESM1_ESM.pdf]

Ghodrati et al “Contrast and luminance adaptation alter neuronal coding and perception of stimulus orientation”

Supplemental information

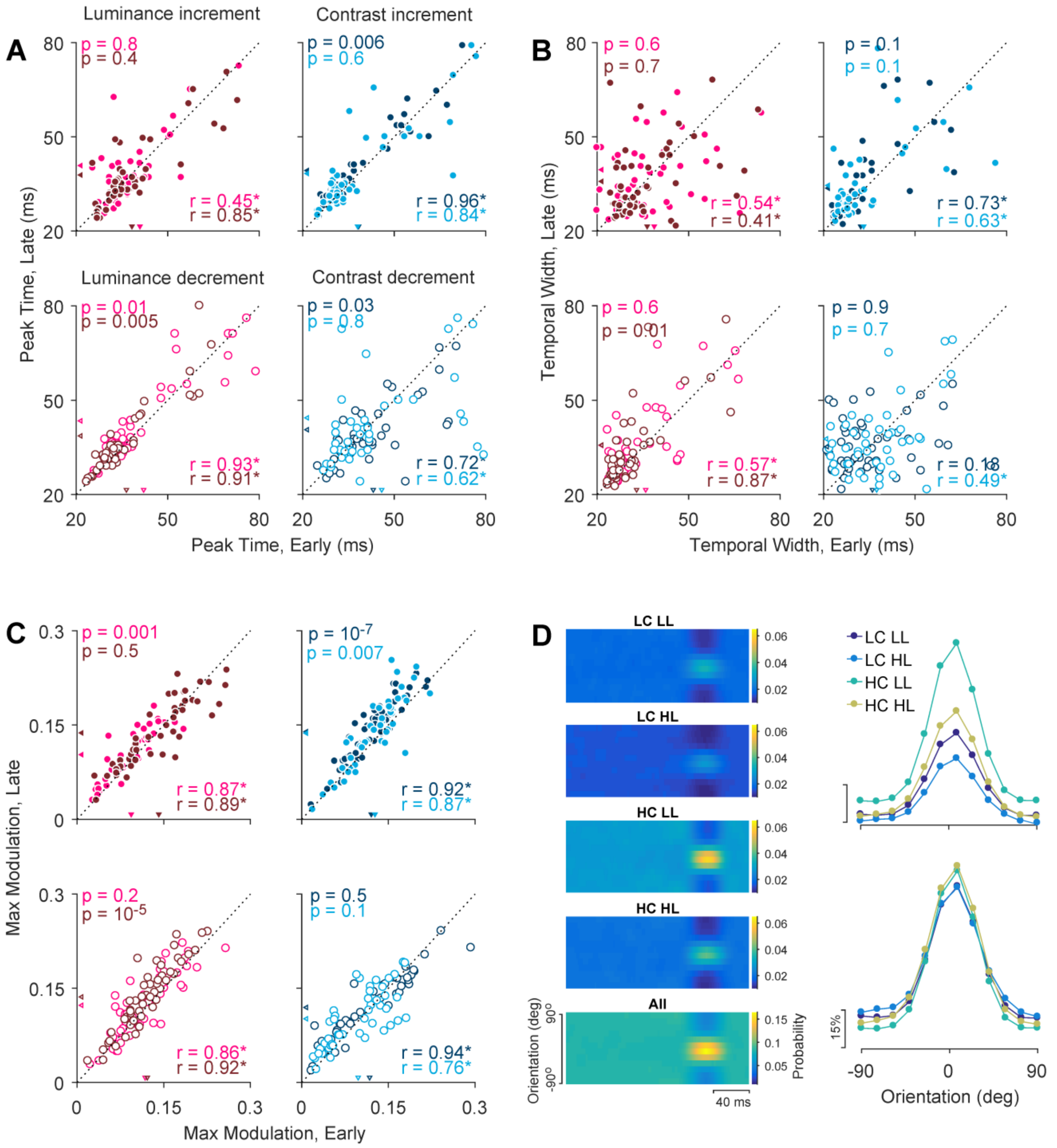

Supplementary Figure 1. Comparing different features extracted from neurons' linear responses to orientation (orientation reverse correlation analysis). Related to Figure 1. Peak time (A), temporal width (B), and maximum modulation (C) in different luminance-contrast switches for 50 highly selective neurons. The solid markers show the results for upward switches (e.g., contrast increment) whole empty markers shows the results for downward switches. (D) Left, reverse correlograms of a sample neuron for every luminance and contrast conditions (see the labels), estimated during the whole 5 s duration (first four heat maps). The last panel illustrates the reverse correlogram estimated from the entire 60 minutes recording, regardless of changes in luminance and contrast of the gratings. Upper-

right, the corresponding tuning functions at peak time for every luminance and contrast conditions. Lower-right, the differences in tuning functions in different conditions can largely be captured by a scaling (gain) factor.

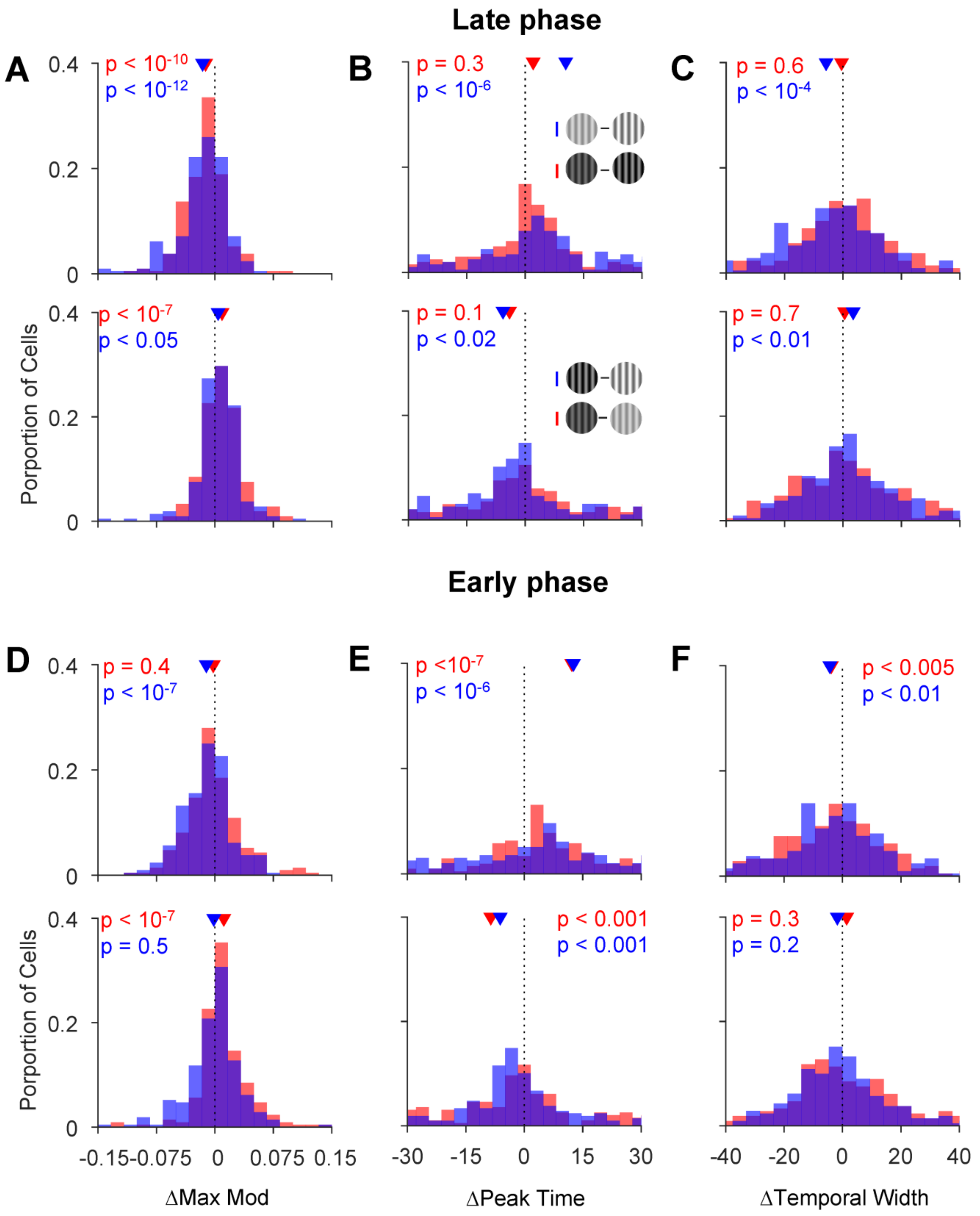

Supplementary Figure 2. Dependence of linear response features on luminance and contrast of the stimuli (between condition comparisons). Related to Figure 1. The distribution of differences in maximum modulation (A), peak time (B), and temporal width (C), in low relative to high contrast (upper panels), and low relative to high luminance (lower panels) during late phase (steady-state). The legends shows how the difference between parameters in different luminance-contrast conditions were calculated. Note that the dash

between two gratings indicates the subtraction. (D-F) As in A-C, but for early phase. n=390, t-test.

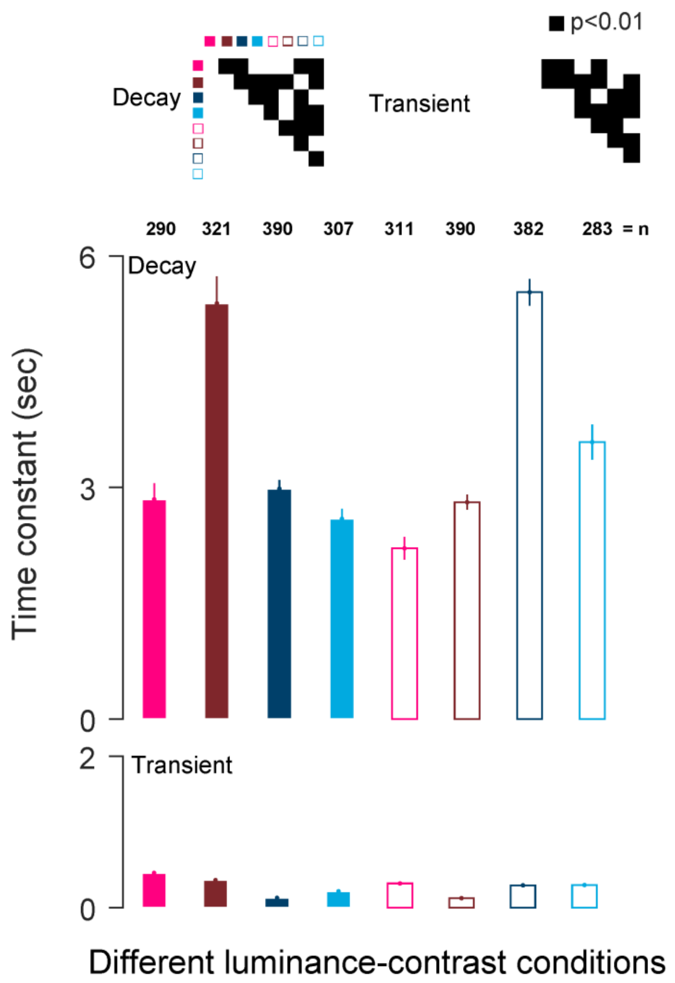

Supplementary Figure 3. Time constant of firing rate adaptation. Related to Figure 3. Lower panel shows the time constants of firing rate fluctuation during transient period (immediately after the switch) while the upper panels indicates the time constants of exponential decay. The solid bars represents the results for upward switches while the empty bar shows the results for downward switches. p-value matrix comparing the time constants in different conditions are depicted at the top, rank-sum test. Error bars are standard error (s.e.m.). Number of neurons are printed on top of bar.

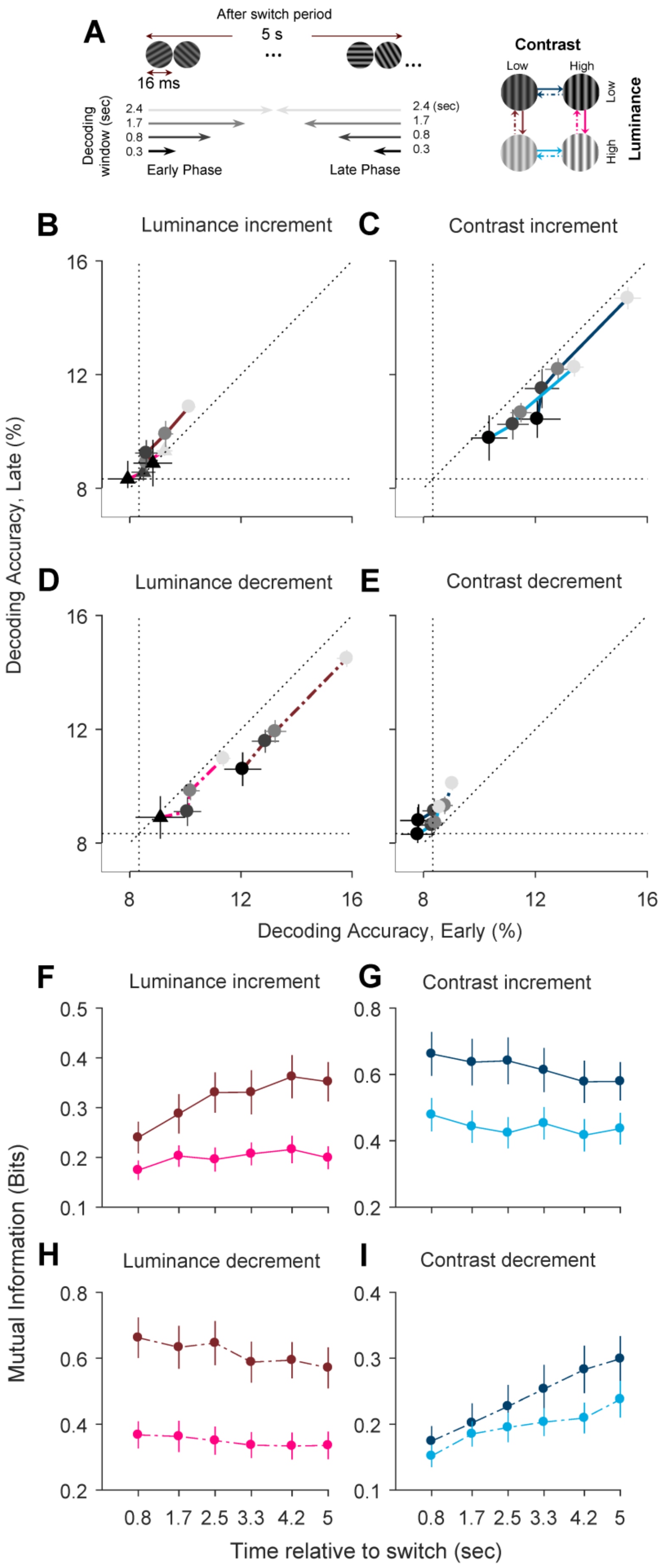

A
After switch period
5 s
16 ms
Decoding window (sec)
2.4
1.7
0.8
0.3
2.4 (sec)
Early Phase
Late Phase
Contrast
Low
High
Luminance
B
Luminance increment
C
Contrast increment
D
Luminance decrement
E
Contrast decrement
Decoding Accuracy, Late (%)
Decoding Accuracy, Early (%)
F
Luminance increment
G
Contrast increment
H
Luminance decrement
I
Contrast decrement
Mutual Information (Bits)
Time relative to switch (sec)

Supplementary Figure 4. Coding of stimulus orientation during adaptation. Related to Figure 2 and Figure 3. (A) Decoding was performed in different time windows during early and late phases after the switch (arrows length printed above indicates window duration). (B-E) Decoding accuracy in different conditions and time windows. The grey level of each data point shows the corresponding time window of decoding (see panel A). Circle data point shows that the decoding performance is significantly above chance (permutation test) and different in early vs late phases or vice versa, whereas triangle data points indicate insignificance. Line colours refer to different upward and downward switches in luminance and contrast. The inset shows the colour code used for each luminance and contrast switch. Decoding algorithm: LDA, number of neurons = 50 randomly selected out of 390 neurons, width of spike counting window, 15 ms, number of random runs = 15. Error bars are standard deviation. SVM classifier provided very similar results. (F-I) Mutual Information between stimulus orientations and neural activity was calculated in six consecutive, non-overlapping time window, each 833 m, during adaptation. Error bars are standard errors, n = 390.

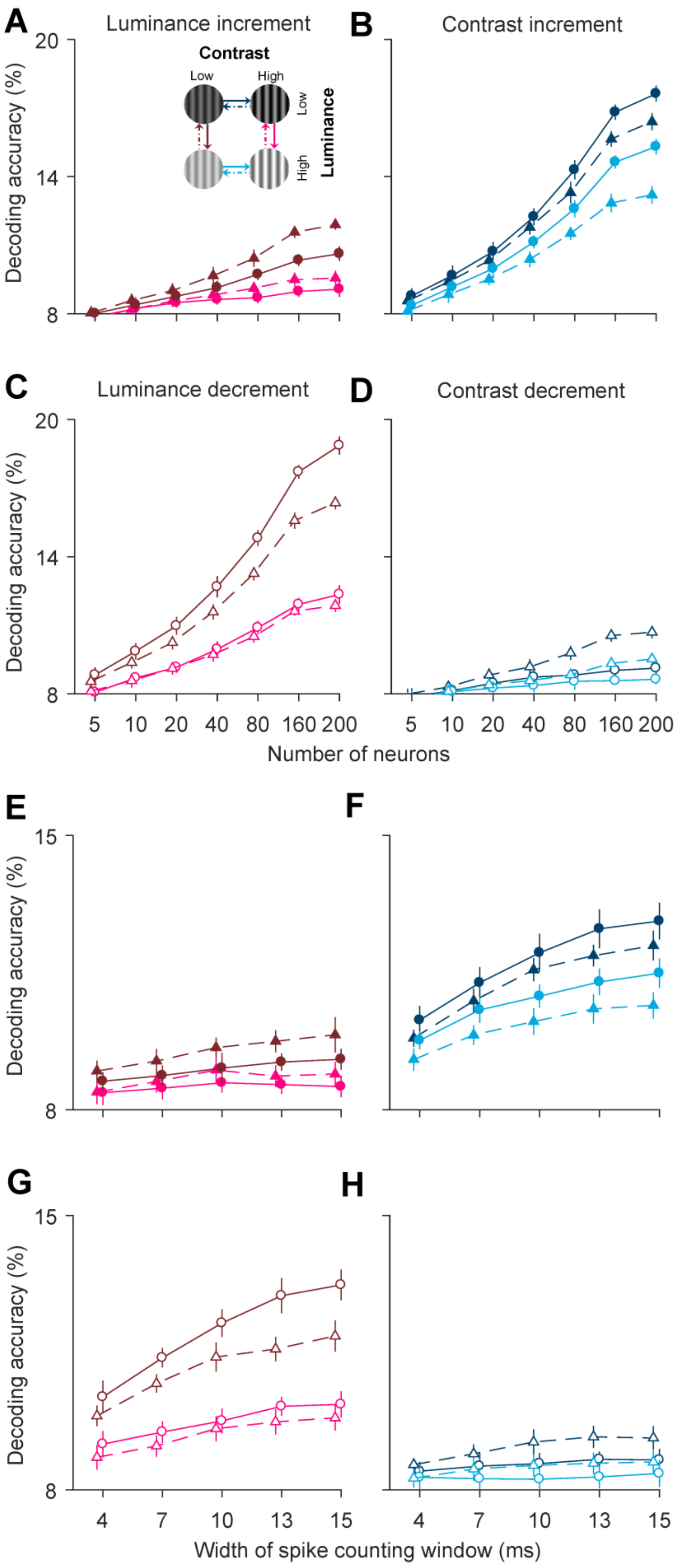
A
Luminance increment
B
Contrast increment
Contrast
Low
High
Low
High
Luminance
C
Luminance decrement
D
Contrast decrement
Decoding accuracy (%)
20
14
8
5 10 20 40 80 160 200
Number of neurons
E
F
G
H
15
8
4 7 10 13 15
Width of spike counting window (ms)

Supplementary Figure 5. The effect of changing the number of neurons (A-D) and width of spike counting window (E-H) on decoding performance in different condition. Related to Figure 3. The solid lines illustrate the decoding accuracy during early phase while the dashed lines indicate the accuracy during late phase. Lines with solid markers indicate the decoding accuracy during upward switches while lines with empty markers show decoding accuracy during downward switches. The inset shows the colour code used for each luminance and contrast switch. As the number of neurons increases, the difference between early and late phase becomes greater (A-D). This can also been seen in most cases when the width of spike counting window increases (E-H). Decoding algorithm: LDA, number of randomised runs = 15. Error bars are standard deviation. SVM classifier provided very similar results. n = 390.

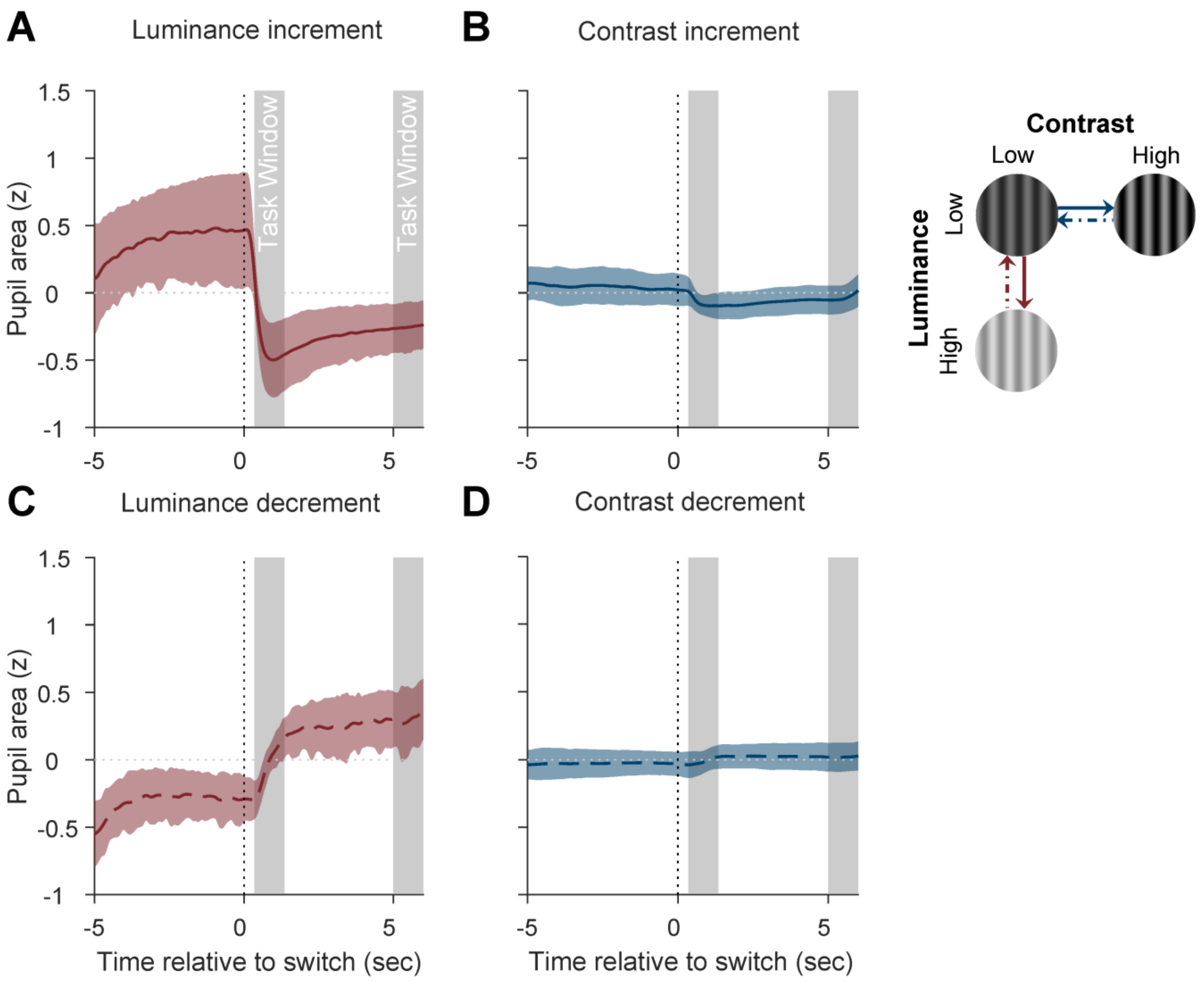

Supplementary Figure 6. Modulation of pupil size during luminance and contrast switches. Related to Figure 5. (A-D) Each trace shows the change in pupil area following a luminance or contrast switch. While luminance switches strongly affect pupil size, contrast switches had minimal effects. The area specified by “task window” is the time window when subjects performed the orientation discrimination task. The traces show the mean (s.e.m.) of pupil area, averaged across all trials for all observers. Pupil size for each observer was normalized to have zero mean and unit standard deviation (z score) across all trials. Number of participants from A-D: n=7, 1, 1, 8.

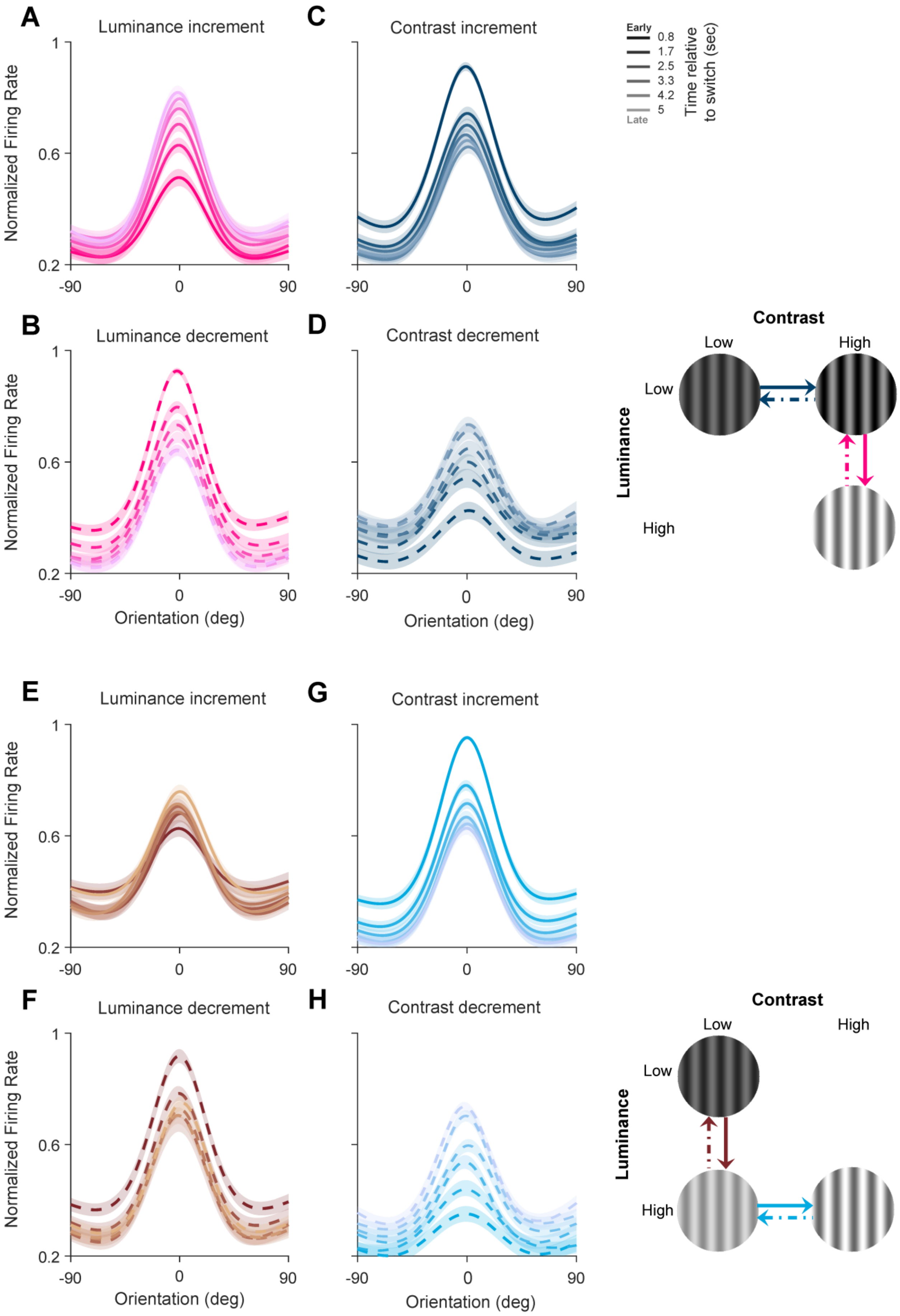
A
Luminance increment
B
Luminance decrement
C
Contrast increment
D
Contrast decrement
Normalized Firing Rate
Orientation (deg)
Early
Late
0.8
1.7
2.5
3.3
4.2
5
Time relative to switch (sec)
Contrast
Low
High
Luminance
E
Luminance increment
F
Luminance decrement
G
Contrast increment
H
Contrast decrement

Supplementary Figure 7. Modulations of orientation tuning during adaptation. Related to Figure 6. (A-H) The average population tuning function of 50 neurons over six consecutive non-overlapping time windows throughout the 5 s adaptation period for different conditions. To calculate the population tuning curve at each time window, we first shifted the preferred orientation of each individual neuron to 0º and then averaged across all neurons. Each panel shows tuning curves following a single switch, as indicated by the insets on the right. We only considered highly orientation-selective neurons for these analyses (n=50). There was strong and systematic multiplicative scaling following luminance increments (A,E) and contrast decrements (D,H), and a combined effect of offset and gain modulations following contrast increments (C,G) and luminance decrements (B,F). This analysis also highlights the different timescales of responses. Following contrast increments and luminance decrements, tuning curve modulations largely occur during the first several hundreds milliseconds after the switch; following luminance increments and contrast decrements there was a more gradual change in the gain of tuning curves. Shaded area is standard error (s.e.m.).
