# Supplementary material for: Contrast and luminance adaptation alter neuronal coding and perception of stimulus orientation
Source: Nat Commun. 2019 Feb 26;10:941. doi: 10.1038/s41467-019-08894-8 (PMC6391449; doi:10.1038/s41467-019-08894-8)
Supplement: Supplementary file 3 — Reporting Summary [file 41467_2019_8894_MOESM3_ESM.pdf]

nature research

Corresponding author(s): Ghodrati, Price

Last updated by author(s): Jan 21, 2019

Reporting Summary

Nature Research wishes to improve the reproducibility of the work that we publish. This form provides structure for consistency and transparency in reporting. For further information on Nature Research policies, see Authors & Referees and the Editorial Policy Checklist.

Statistics

For all statistical analyses, confirm that the following items are present in the figure legend, table legend, main text, or Methods section.

n/a	Confirmed	
☐	☒	The exact sample size (*n*) for each experimental group/condition, given as a discrete number and unit of measurement
☐	☒	A statement on whether measurements were taken from distinct samples or whether the same sample was measured repeatedly
☐	☒	The statistical test(s) used AND whether they are one- or two-sided<br>*Only common tests should be described solely by name; describe more complex techniques in the Methods section.*
☒	☐	A description of all covariates tested
☐	☒	A description of any assumptions or corrections, such as tests of normality and adjustment for multiple comparisons
☐	☒	A full description of the statistical parameters including central tendency (e.g. means) or other basic estimates (e.g. regression coefficient) AND variation (e.g. standard deviation) or associated estimates of uncertainty (e.g. confidence intervals)
☐	☒	For null hypothesis testing, the test statistic (e.g. *F*, *t*, *r*) with confidence intervals, effect sizes, degrees of freedom and *P* value noted<br>*Give P values as exact values whenever suitable.*
☒	☐	For Bayesian analysis, information on the choice of priors and Markov chain Monte Carlo settings
☒	☐	For hierarchical and complex designs, identification of the appropriate level for tests and full reporting of outcomes
☐	☒	Estimates of effect sizes (e.g. Cohen's *d*, Pearson's *r*), indicating how they were calculated

Our web collection on statistics for biologists contains articles on many of the points above.

Software and code

Policy information about availability of computer code

Data collection	The data sets for the current study are available from the corresponding author on request.
Data analysis	MATLAB code used for the analysis is available from the corresponding author on request.

For manuscripts utilizing custom algorithms or software that are central to the research but not yet described in published literature, software must be made available to editors/reviewers. We strongly encourage code deposition in a community repository (e.g. GitHub). See the Nature Research guidelines for submitting code & software for further information.

Data

Policy information about availability of data

All manuscripts must include a data availability statement. This statement should provide the following information, where applicable:

- Accession codes, unique identifiers, or web links for publicly available datasets
- A list of figures that have associated raw data
- A description of any restrictions on data availability

The data sets for the current study are available from the corresponding author on request. MATLAB code used for the analysis is available from the corresponding author on request.

Field-specific reporting

Please select the one below that is the best fit for your research. If you are not sure, read the appropriate sections before making your selection.

☒ Life sciences ☐ Behavioural & social sciences ☐ Ecological, evolutionary & environmental sciences

Life sciences study design

All studies must disclose on these points even when the disclosure is negative.

Sample size	No statistical methods were used to pre-determine sample sizes. Our sample sizes (animal and neuron numbers) are similar to or greater than those reported in previous comparable publications.
Data exclusions	We analysed those neurons that showed significant orientation selectivity. Neurons were deemed to be orientation selective if they satisfied three criteria: (1) maximum probability (probability at peak time) in the reverse correlation analysis exceeded 3×STD of the baseline probability; (2) the von Mises function fit (Equation 1) was significantly better than a flat line (F-test, $p < 0.05$); (3) the bandwidth of von Mises function fit was 20–95°. Across all recordings (511 isolated neurons), 453 neurons were visually responsive, and of these, 390 neurons (86%) were orientation selective when tested with at least one of the luminance-contrast combinations. A neuron was selected as visually responsive if its spiking activity significantly increased above baseline after stimulus presentation based on standard forward correlation stimuli.<br>For human behavioral experiment. The data of two subjects were excluded because their psychometric curves were flat at 50%, indicating that they were guessing or did not understand the task.
Replication	We recorded the data from three different monkeys and found similar results in every animal, suggesting the reproducibility of the findings.
Randomization	This is not relevant to our study as we are not comparing two different cohorts. Each neuron was tested with multiple stimulus types.
Blinding	Stimulus presentation order was generated randomly and not under experimenter control. No other blinding was performed during analysis.

Reporting for specific materials, systems and methods

We require information from authors about some types of materials, experimental systems and methods used in many studies. Here, indicate whether each material, system or method listed is relevant to your study. If you are not sure if a list item applies to your research, read the appropriate section before selecting a response.

Materials & experimental systems

n/a	Involved in the study	
☒	☐	Antibodies
☒	☐	Eukaryotic cell lines
☒	☐	Palaeontology
☐	☒	Animals and other organisms
☐	☒	Human research participants
☒	☐	Clinical data

Methods

n/a	Involved in the study	
☒	☐	ChIP-seq
☒	☐	Flow cytometry
☒	☐	MRI-based neuroimaging

Animals and other organisms

Policy information about studies involving animals; ARRIVE guidelines recommended for reporting animal research

Laboratory animals	Three adult male marmoset monkeys (Callithrix jacchus).
Wild animals	The study did not involve wild animals
Field-collected samples	The study did not involve samples collected from the field
Ethics oversight	Experiments were conducted in accordance with the Australian Code of Practice for the Care and Use of Animals for Scientific Purposes, and all procedures were approved by the Monash University Animal Ethics Experimentation Committee .

Note that full information on the approval of the study protocol must also be provided in the manuscript.

Human research participants

Policy information about studies involving human research participants

Population characteristics	In total, we recorded behavioural data from 31 subjects (16 females, age 20–34 years, normal or corrected-to-normal vision). The data of two subjects were excluded because their psychometric curves were flat at 50%, indicating that they were guessing or did not understand the task. Note that some subjects participated in more than one of the four luminance and contrast tasks.
Recruitment	Subjects (n=31 see above) were students from Faculty of Medicine, Nursing and Health Sciences at Monash University. All subjects voluntarily participated in the experiments and gave their written consent prior to participation.

Ethics oversight	All human psychophysical experiments were conducted in accordance with the The National Statement On Ethical Conduct In Human ResearchAustralian Code of Practice for the Care and Use of humans for Scientific Purposes, and all procedures were approved by the Monash University hHuman Research Ethics Experimentation Committee.

Note that full information on the approval of the study protocol must also be provided in the manuscript.
